# Fueling Clinical and Translational Research in Appalachia: Informatics Platform Approach

**DOI:** 10.2196/17962

**Published:** 2020-10-14

**Authors:** Alfred A Cecchetti, Niharika Bhardwaj, Usha Murughiyan, Gouthami Kothakapu, Uma Sundaram

**Affiliations:** 1 Department of Clinical and Translational Science Joan C. Edwards School of Medicine Marshall University Huntington, WV United States

**Keywords:** Appalachian region, medical informatics, health care disparities, electronic health records, data warehousing, data mining, data visualization, machine learning, data science

## Abstract

**Background:**

The Appalachian population is distinct, not just culturally and geographically but also in its health care needs, facing the most health care disparities in the United States. To meet these unique demands, Appalachian medical centers need an arsenal of analytics and data science tools with the foundation of a centralized data warehouse to transform health care data into actionable clinical interventions. However, this is an especially challenging task given the fragmented state of medical data within Appalachia and the need for integration of other types of data such as environmental, social, and economic with medical data.

**Objective:**

This paper aims to present the structure and process of the development of an integrated platform at a midlevel Appalachian academic medical center along with its initial uses.

**Methods:**

The Appalachian Informatics Platform was developed by the Appalachian Clinical and Translational Science Institute’s Division of Clinical Informatics and consists of 4 major components: a centralized clinical data warehouse, modeling (statistical and machine learning), visualization, and model evaluation. Data from different clinical systems, billing systems, and state- or national-level data sets were integrated into a centralized data warehouse. The platform supports research efforts by enabling curation and analysis of data using the different components, as appropriate.

**Results:**

The Appalachian Informatics Platform is functional and has supported several research efforts since its implementation for a variety of purposes, such as increasing knowledge of the pathophysiology of diseases, risk identification, risk prediction, and health care resource utilization research and estimation of the economic impact of diseases.

**Conclusions:**

The platform provides an inexpensive yet seamless way to translate clinical and translational research ideas into clinical applications for regions similar to Appalachia that have limited resources and a largely rural population.

## Introduction

### Background: Unique Challenges in Appalachia

With regard to health care, Appalachia with its predominantly rural communities is known to have one of the worst outcomes in the United States [1]. This is especially true of southern and central rural Appalachia, which face some of the most severe health disparities in the nation [1]. Over the years, the gap in the overall health between Appalachia and the nation as a whole has continued to grow [2,3]. To close this gap, it is critical to identify the cause of these disparities and direct efforts toward developing necessary interventions to address them.

Such an effort necessitates the adoption of modern technologies such as a centralized research data warehouse to house all data necessary to obtain a comprehensive picture of the health of the Appalachian population before analysis to gain actionable insights can be performed. A centralized data warehouse, once considered strictly a business tool, has evolved into an important instrument for cost containment, tracking of patient outcome, providing clinical decision support at the point of care, improving prognostic accuracy, and facilitating research [4]. Thus, rural academic medical centers have moved toward implementing data warehouse systems that feed analytical systems for research needs [5]. This entails (1) the integration of data from different types of medical settings (ie, multi-institutional) such as hospitals, clinics, and specialty centers; (2) linkage of financial data with clinical data—a well-established practice proven to be pivotal to high-quality care and great economic outcomes [6,7]; and (3) integration of other determinants of health such as environmental [8], social [9], and spiritual factors [10] to create longitudinal health records across the care continuum.

However, there are challenges in creating a multi-institutional data warehouse [11]. The electronic health records (EHRs) do not easily interact with one another due to the use of nonstandard terminologies and difficulty in understanding the flow of information. In addition, significant differences exist between rural and urban health systems [12-16]. Unlike their urban counterparts, health care data in Appalachia are typically fragmented, existing in silos within dissimilar databases, registries, data collections, and departmental systems. With innovations in medical technology, the list of data sources continues to grow, producing unprecedented amounts of data from all aspects of care, including diagnosis, medication, procedures, laboratory test results, imaging data, and patient self-monitoring [17-21]. To complicate matters, the overall health and health behaviors of Appalachians are strongly affected by Appalachia’s unique culture, geography, and health system issues [22-24]. Consequently, Appalachian academic medical centers face the complex challenge of collecting, organizing, standardizing, and analyzing these enormous quantities of heterogeneous data originating from a wide variety of sources to address the unmet needs of the population they serve.

### Why an Informatics Platform?

Data integration and interoperability have been shown to be key to unlocking these data for data analytics, enabling the development of novel patient management strategies for rural hospitals [25,26] and translational research that leads to new approaches at the bedside for prevention, diagnosis, and treatment of disease, which are essential to improving the health of a population [27-29]. Data analytics, once the domain of the statistician, has now become an equal partner in clinical research and research operations [30,31]. Following the data explosion, data analytics increasingly involves the use of visual analytics tools such as Tableau (Tableau Software Inc) and Power BI (Microsoft Corp) to explore data easily and in a self-service fashion and to clearly and effectively communicate complex ideas [32], especially to those members of the medical community who might not have an intimate understanding of the underlying data. Furthermore, machine learning is gaining importance, especially in the area of predictive analytics, to improve the practice of medicine and to infer potentially innovative risk factors [28,33-35].

However, these applications (eg, data warehouse, data analytics, statistical analysis, machine learning, visual analytics) are generally uncoordinated without any overarching governance. Thus, we developed an informatics platform, that is, a suite of interconnected, coordinated applications hosted within an operational environment [36], called the Appalachian Informatics Platform, in West Virginia—the only state located entirely in Appalachia—that facilitates interoperable access to integrated information, data visualization, and data analytics, thereby functioning as an excellent basis for clinical and translational research to improve health care.

The goal of this study is to describe the structure and process of development of the Appalachian Informatics Platform and demonstrate its value in supporting clinical and translational research.

## Methods

**Figure 1:**
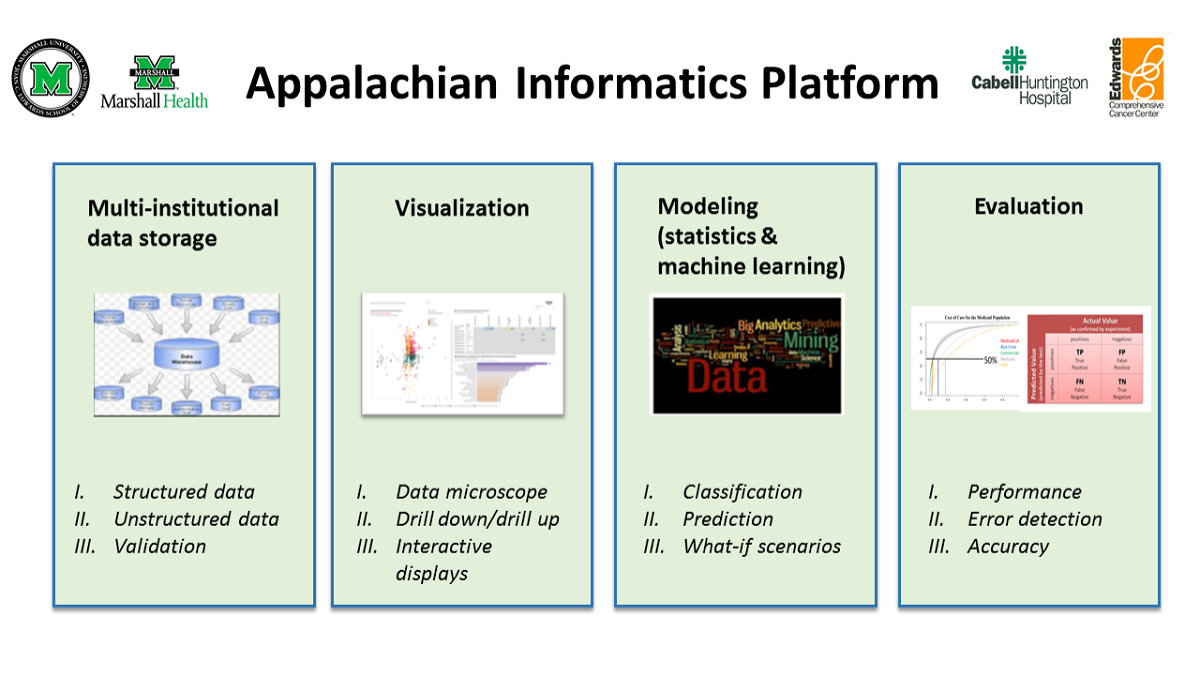
Appalachian informatics platform.

The Appalachian Informatics Platform (Figure 1) is composed of 4 major components: (1) multi-institutional data storage—clinical data warehouse (CDW); (2) modeling (statistical and machine learning); (3) visualization; and (4) evaluation. Each of these components is described in detail in separate sections.

The CDW forms an integral part of the Appalachian Informatics Platform. The Appalachian Informatics Platform, in addition to the CDW, contains embedded data analytics (modeling and evaluation) and interactive visualization tools (eg, Tableau [Tableau Software Inc], Power BI [Microsoft Corp]). Together, these enable the analysis of Appalachian health information to speed up the transition of translational research ideas into clinical practice.

The CDW serves as a secure source of quality data for descriptive, diagnostic, predictive, and prescriptive analytics for research and operational needs. The visual analytics tools enable an initial exploratory analysis of the processed data and the interactive presentation of analytical findings for further analysis and review. Depending on the use case, data can be analyzed using statistical modeling via external (eg, SPSS [IBM Corp], Stata [StataCorp]) or integrated (eg, R [R Foundation for Statistical Computing], Python [Python Software Foundation] in Structured Query Language [SQL]) applications or machine learning modeling. The performance of the resulting models was evaluated using appropriate metrics. Once trained and evaluated, machine learning models can be deployed and stored in the CDW for future use if needed. Furthermore, the stored machine learning models can be continuously evaluated and improved as more data are generated.

The informatics committee governs the access to and utilization of the Appalachian Informatics Platform and ensures adherence to security and privacy rules. In addition, team-building activities are also incorporated into our clinical informatics model to foster the development of an effective clinical informatics team.

### Multi-Institutional Data Storage: Appalachian Clinical and Translational Science Institute-Clinical Data Warehouse

The Appalachian Clinical and Translational Science Institute (ACTSI)’s Division of Clinical Informatics solicited buy-in from different entities, namely, Cabell-Huntington Hospital (CHH), Edwards Comprehensive Cancer Institute (ECCC), Marshall Health (MH) practice plan, and Marshall University Joan C Edwards School of Medicine (MU JCESOM), to build the Appalachian Clinical and Translational Science Institute-Clinical Data Warehouse (ACTSI-CDW) in West Virginia. An agreement was created between these entities that provided access to both financial and clinical data.

The multi-institutional CDW contains more than 9 years of billing and clinical data. It comprises relational tables and dimension and fact tables (Online Analytical Processing [OLAP] cube), which enable secure data storage and data access. Designed from the start to facilitate information flow, the CDW can send out a stream of near real-time data that can be used for any authorized research purpose. Documentation includes a data dictionary and flowcharts. Flowcharts follow the patient from admission (or appointment, if outpatient) to discharge (or exit, if outpatient). The data dictionary contains the standardized and source field names, descriptions, and properties along with the associated metadata for the data contained within the data warehouse. For instance, (1) the entry of a patient into any medical service (admission or appointment) was combined with the single term *encounter* and (2) a higher level of precision was introduced by separating patient age into 2 variables, current age or the age when the procedure was performed.

The CDW process is based on an older data warehouse process developed at the University of Pittsburgh [37]. The process is as follows:

1. Data dictionaries are created by recording institutional source field names and field properties and linking them to the standardized CDW names and properties found within the CDW databases. Descriptions of each field (source and CDW) are included.
2. Individual institutional flowcharts show the workflow of the data and the location of the people responsible for the quality of the data, which are also used for quality control purposes.
3. At present, the CDW contains data from 6 institutional software packages hosted in various parts of the country (eg, Cerner data from Kansas City, Missouri; McKesson data from North Druid Hills, Georgia; etc). The data are exported in a standard format (ie, ASCII flat file, XML, etc) and transferred through secure file transfer protocol (eg, Cerberus [Cerberus, LLC]) to the CDW Development server.
4. The data are integrated into the Microsoft SQL databases using Microsoft SQL Server Integration Services (SSIS), a graphical tool that extracts, transforms, and loads (ETL) the data to target schemas that will be used to contain the target data objects: relational tables, dimensions, and cubes. ETL systems enable a smooth migration from one system to another irrespective of the underlying storage system.
5. Conformed dimensions were developed, and patient linkages using various methods (eg, simple heuristics) [38] were also available and made at this time.
6. At present, a transactional grain fact table has been developed, but other fact tables will be created as needed.
7. The CDW contains internal structured billing and EHR data (ie, demographics, encounter details, vitals, medications, procedures, diagnoses, orders, immunizations, laboratory and imaging results, date and time, payee, and provider). It also contains unstructured EHR data (eg, H&P, admission notes, discharge summaries, other clinical notes). These data are received from MH, CHH, and MU JCESOM’s ECCC as well as from other outside institutions. In addition, non-EHR data are incorporated using REDCap.
8. Unstructured data are analyzed using text analytics tools, and classification variables based on text mining are incorporated into the CDW.
9. The data structure (OLAP cubes and relational tables), once checked and verified, is transferred from the secure development server to the secure production server for use.
10. Various security measures (eg, IP and password restrictions) are in place to prevent unauthorized use.
11. The CDW structure, which stores multi-institutional medical information, can now provide data for both operational and research analytical model development (statistical or machine learning) using very simple deidentified interfaces (eg, Excel [Microsoft Corp]) or more complex interactive tools (eg, R [R Foundation for Statistical Computing], Tableau [Tableau Software Inc], Power BI [Microsoft Corp], etc). Within the CDW, the data can be manipulated, cleaned, and prepared before the analysis as needed.
12. Structured and unstructured data currently exist within the CDW. Image and BioSample data will soon be incorporated (like the Pittsburgh model), but the full design has not been finalized yet. An *Honest Broker* person assumes control of sample shipping and receiving.
13. Standard Operation Procedures have been developed for administrative and technical areas.
14. The Health Insurance Portability and Accountability Act (HIPAA) guidelines are followed, and protocol to protect patient information has also been implemented.

The CDW is contained within a Microsoft SQL database that can interact with outside objects using other electronic methods such as SignalR, a software library for Microsoft ASP.NET that allows server code to send asynchronous notifications to client-side web applications and SqlDependency, an object that represents a query notification dependency between an application and an instance of SQL server. Objects such as these provide the ability for the data warehouse to interact in real time with the outside regional population using the newest technologies such as Microsoft Machine Learning Server with embedded R or Python procedure coding.

#### Data Validation

The information derived from multiple data sources can have inconsistencies and missing values because of their heterogeneous nature that needs to be corrected [39-42]. Thus, for each research study, clinical and translational researchers using the data warehouse are required to verify a random sample (calculated on the basis of the size of the study population) of all extracted study data are directly verified at the original data source to ensure data accuracy and validity. Identified errors or omissions are transmitted back to the host systems for correction or inclusion.

#### Augmenting the CDW Using REDCap

For certain studies, data available in the CDW may not be precise enough or include variables needed to perform this study. For such studies, data can be augmented using data capture tools. One such tool is the Research Electronic Data Capture, or REDCap, a workflow methodology and software solution designed for the rapid development and deployment of electronic data capture tools to support clinical and translational research [43-45].

Our institution has deployed and maintains 2 REDCap servers: secure (located under institutional firewall) and global (outside the firewall). The secure REDCap system is used for storing data considered protected health information (PHI) under HIPAA. The global system, on the other hand, is used to store deidentified or non-PHI data. These data are then transferred to and stored within the multi-institutional data warehouse. This method of augmenting the information pulled from the existing source systems provides research-grade data from outside sources that are normally not contained within a data warehouse.

### Visualization

Visualization of information is an excellent method of providing knowledge that can be easily understood by any member of the health care discipline. Within the informatics platform, Tableau provides interactive drill-down and drill-up capabilities for specific projects.

Tableau is a visual analytics tool that provides an interactive method of exploring multidimensional data, optimized from the data warehouse and OLAP data sources. Tableau, using either indexed relational tables or a data cube, can perform associated operations such as slice, dice, roll-up, and drill-down on the data, providing detailed interactive visual overlays that range from the lowest grain of the data to high-level representations of the data. Tableau charts, graphs, filters, and maps can provide visualization of the various subgroups of interest using a storyboard approach that presents a specific question followed by an interactive dashboard that explores that question in detail. The use of visual elements such as logos, pictograms, icons, or pictures into the dashboards, in association with the subgroups, provides easy-to-reference image aids that provide clarity and understanding of complex information. The data warehouse provides the drill-down, drill-up and slice and dice capability, whereas the hub design connects both financial and clinical data to provide a full picture.

The developed interactive dashboards are securely shared with users within a department or a team, as needed, through the use of Tableau Server [46].

### Modeling (Statistics and Machine Learning)

The modeling component of the informatics platform supports the construction of tailored regional models (statistical or machine learning) to understand and predict disease and other medical events within this region. EHR is primarily a billing system, research only being a secondary function and, thus, is heterogeneous, incomplete, and noisy [25], leading to unrepresentative samples, selection bias, and misclassification [47]. During the modeling process, these issues are eliminated or minimized.

To assist in modeling, software packages such as Stata [StataCorp] and SPSS [IBM Corp] and embedded open-source machine learning programs (eg, R [R Foundation for Statistical Computing], Python [Python Software Foundation]) are used. This enables faster and easier development of classification, regression, and clustering algorithms for research use. In addition, we utilize products such as Microsoft’s LINQ to electronically gather information and directly incorporate that information into the CDW.

### Evaluation

During the modeling process, evaluation of the data set as it relates to the regional population is carried out. Local experts native to this region are asked to evaluate the model from a clinical as well as a financial standpoint. Poverty is endemic within the Appalachian population, and a model that suggests the use of a very expensive medication or procedure over an older but less expensive medication or procedure is unlikely to be used [48]. Thus, the model must take into account whether the patient has the means and access to the recommended medication or procedure [49]. In addition, the willingness of Appalachian medical institutions and health care providers to follow the model’s suggestions must also be evaluated.

Once developed, the models were tuned and tested. Location, time of treatment, outside temperature, and other contributory factors available within the CDW were employed to fine-tune the models, as applicable. The performance of the models was measured using the R programming environment using measures such as area under curve, sensitivity, specificity, *F*_1_ score, precision, recall, etc.

### Security, Privacy, and the Informatics Committee

Data access and usage are permitted only as described in the mutual agreement between the 3 institutions and are subject to internal security and privacy rules. All data requests must follow the standard operating procedure built on the basis of mutual multi-institutional agreement. Foremost, the researcher must have appropriate credentials and authorization to be able to request for data. If the researcher is authorized to make requests, he or she must obtain the IRB approval for his or her proposed study and submit the IRB proposal and supporting documentation for review by the informatics committee. The informatics committee, independent of the IRB, reviews all requests for data from the data warehouse to ensure compliance with the agreement. If the research project is approved, the research team designated members are scheduled for the deidentified data extraction process.

### Team Building

Integral to the informatics platform is team building that builds upon previous work [37]. To facilitate effective team meetings and interprofessional collaboration (local and global) without the need or expense of constant travel, a permanent clinical informatics conference room with a fixed connected computer, an uninterruptable power supply (UPS), a smart board, a camera, and a speaker system, along with a video conferencing system (Zoom) connectivity, was built. This ensures adequate communication among all those involved (ie, team members, users, leadership, etc) and access to resources that would otherwise be unavailable.

## Results

Since the implementation of the platform, several studies have been conducted. Each study listed below was approved by the informatics committee, and the deidentified data and platform tools were made available securely to the research team.

To evaluate the functionality and value of this platform, we first analyzed the aggregated data of Medicaid-insured patients across different health systems using the interconnected applications within the platform for population health management. Relevant data were extracted from the CDW, followed by exploratory analysis using a Tableau dashboard. Due to the isolated nature of the study population, regional variables such as distance from the CHH and weather conditions (ie, temperature) were also included. Errors and missing values were identified using the dashboard, and data were subsequently cleaned and prepared. Using these clean data, the regional population was classified into 3 spend categories: *low cost, acute*, and *persistent* subgroups on the basis of the charges accrued. Next, the Charlson Comorbidity Index (CCI) was incorporated into the CDW to predict mortality risk within 1 year of hospitalization for patients with comorbid conditions within each spend category (Table 1) [50,51]. Of these categories, the persistent group had the largest percentage of patients with a high risk of mortality, followed by acute and low cost after excluding the deceased patients (persistent: 898/1247, 72.01%; acute: 2074/6946, 29.86%; low cost: 5130/102,814, 4.99%). The CCI was not very sensitive in predicting the risk of mortality but was very specific and accurate (sensitivity: 896/1512, 59.26%; specificity: 102,905/111,007, 92.7%; accuracy: 103,801/112,519, 92.25%). The effect of distance and weather on the CCI needs further investigation that is being conducted. Adjustments are being made to this standard national index to incorporate other Appalachian characteristics that could improve the sensitivity of this risk scoring system.

This way, the platform has been utilized for a variety of purposes such as increasing knowledge of the pathophysiology of diseases, risk identification, risk prediction, health care resource utilization research, and estimation of the economic impact of diseases to enable data-driven clinical decisions, leading to improved clinical outcomes. Textbox 1 contains a list of studies conducted so far.

**Table 1:** The 10-year mortality risk predicted using the Charlson Comorbidity Index.

Mortality risk	Deceased, n (%)	Alive, n (%)
High risk	896 (0.80)	8102 (7.20)
Low risk	616 (0.55)	102,905 (91.46)

**Studies conducted using the Appalachian Informatics Platform.**

Diagnostic accuracy improvement studies

- Albumin Level as a Risk Marker and Predictor of Peripartum Cardiomyopathy [52]
- Clinical Determinants of Myocardial Injury, Detectable and Serial Troponin Levels Among Patients With Hypertensive Crisis [53]
- Is Fever a Red Flag for Secondary Bacterial Pneumonia During RSV Bronchiolitis [54]
- Metabolic Syndrome: Are Current Colon Cancer Screening Guidelines Enough in a Rural Population? [55]
- Utilization of Appalachian Clinical and Translational Science Institute Data Warehouse to More Accurately Predict Disease Processes Important for Central Appalachia [56]

Resource utilization and financial impact research studies

- Fueling Dementia Research in Appalachia via Appalachian Informatics Platform: A Longitudinal Study [57]
- Hospital Emergency Department Visits For Non-Traumatic Oral Health Conditions [58]

Studies to understand disease pathophysiology

- Serum Calcium Homeostasis and Volume Dynamics in Alzheimer’s Disease and Diabetes Mellitus-2 [59]

Five studies utilized the platform for risk identification and risk prediction to improve diagnostic accuracy [52-56]. Sundaram et al [56] demonstrated the value of ACTSI-CDW as a primary source to improve the diagnosis of metabolic syndrome, a diagnosis very relevant to the Central Appalachian population. The researchers discovered that utilizing billing codes alone severely underestimated the number of patients with metabolic syndrome by a factor of more than 10 as compared with looking at specific criteria that determine this diagnosis [56]. Another study assessed the relationship between metabolic syndrome and colorectal cancer and found that patients with metabolic syndrome, especially those with insulin resistance, were more likely to have colorectal cancer, indicating the probable need for earlier screening for colorectal cancer in these patients [55]. Elmore et al [54] examined the role of fever in predicting the development of secondary bacterial pneumonia in children with RSV and other viral illnesses. They found that febrile children were 2 to 8 times (RSV, 47/78 vs 27/100; other bronchiolitis, 54/83 vs 7/88) more likely to have secondary bacterial pneumonia compared with afebrile children and, thus, may need to be aggressively evaluated to enable early diagnosis and treatment [54]. Amro et al [52] studied the relationship between hypoalbuminemia and peripartum cardiomyopathy and noted that lower albumin levels were significantly associated with peripartum cardiomyopathy (*P*<.001; odds ratio 0.033, 95% CI 0.034-0.865) and could potentially be used as a risk marker for it. Acosta et al [53] used data from the ACTSI-CDW to identify risk factors (lower BMI, before CHF, and prior use of aspirin) that predict myocardial injury, detectable troponin, and increase in serial troponin levels in patients with hypertensive crisis.

Ferdjallah et al [59] analyzed the data from the ACTSI-CDW to understand how Alzheimer disease and diabetes mellitus affect serum calcium homeostasis and extracellular fluid volume. They observed that acute changes in serum calcium were significantly correlated with changes in extracellular fluid volume in both disease states [59].

**Figure 2:**
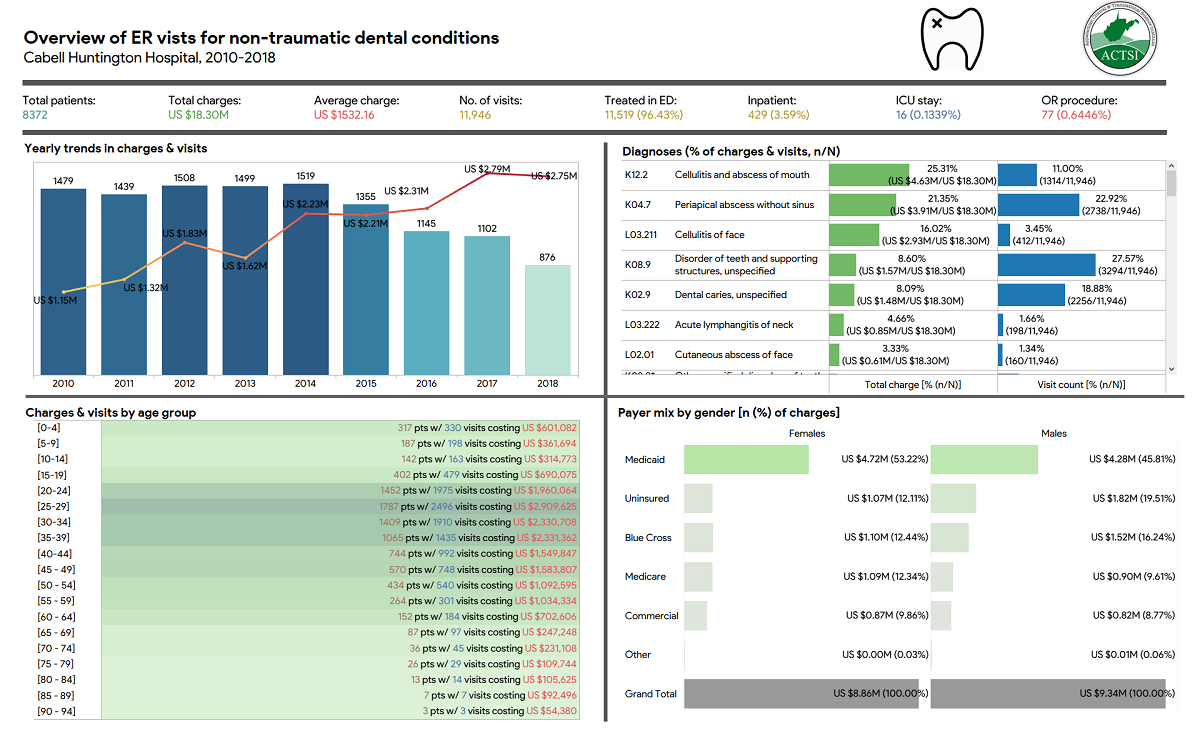
Tableau dashboard displaying patterns and trends in charges for non-traumatic dental ER visits at Cabell Huntington Hospital between 2010 and 2018. ER: emergency room.

The platform has also been applied in 2 studies to assess resource utilization (eg, emergency room, medications, etc) and the financial impact of the disease. For instance, Bhardwaj et al [57] utilized the platform to identify the problems associated with benzodiazepine use in geriatric patients within the health system, such as a higher number of emergency room visits and charges in geriatric patients with dementia plus at least one BZD prescription. In another study [58] that aimed to measure the volume and cost of emergency room use for these conditions and identify the factors that predict such use, the researchers built a dashboard (Figure 2) to easily explore and analyze relevant data on nontraumatic dental conditions that led to emergency room visits and to report the key findings of the study. The authors [58] observed that emergency room visits by uninsured patients were 4 times more likely and those by Medicaid insured 2 times more likely to be for dental problems than Medicare-insured patients.

## Discussion

### Utility of the Appalachian Informatics Platform

The Appalachian Informatics Platform has supported several research projects involving the use of different components of the platform, depending on project needs. The studies described reported findings that are seldom reported in this region, enhanced our knowledge of pathophysiology and risk factors, and helped estimate and analyze resource utilization and economic burden of certain diseases within Appalachia using minimal resources (a small IT team and a relatively inexpensive platform).

Before the implementation of the platform, many research studies that followed the patient across multiple care settings or involved analysis of big data were not possible due to the unavailability of technical and economic resources owing to a lack of buy-in from rural health care organizations. As the data existed in silos, there was a lack of standardization and normalization, which resulted in major data inconsistencies. Studies conducted using these disjointed data sets often used unrepresentative small biased samples and had low statistical power and quality.

The introduction of the platform has helped address these issues. It is now easier to pinpoint and correct errors and/or missing values and understand the distribution of data using visual analysis tools. Further, the time needed to conduct these studies from start to finish has been greatly reduced owing to the availability of all applications necessary to complete the study within the platform. This has been specifically useful because many researchers do not have the technical skills needed to perform complex and advanced data analysis, especially on larger data sets.

The paper also revealed that national models do not necessarily perform well when applied to the Appalachian population. The Appalachian Informatics Platform allows for seamless integration of regional variables into the national model, which may improve the performance of these models. For each of the top 10 causes of death in West Virginia in 2017 per the Centers for Disease Control and Prevention [60], a machine learning algorithm was used to predict outcomes on a national level: heart disease [61,62], cancer [63,64], accidents [65,66], respiratory disease [67,68], stroke [69,70], diabetes [71,72], Alzheimer disease [73,74], pneumonia [75,76], kidney disease [77,78], and suicide [79,80]. Each of these cited models could be modified to fit the characteristics of the Appalachian population, especially those characteristics that make this region different in terms of geography, economy, education, and culture from the rest of the United States. The development of these regional models could help rural health general practitioners tackle complex medical conditions without the need for an expensive specialized health care provider nearby [46].

We hope that this paper will help other rural health care organizations, such as ours, that serve underserved populations realize the value and ease of using an informatics platform to conduct research and improving care for their patients despite limited resources.

### Ongoing Projects and Future Directions

At present, a model that utilizes embedded data analytics to monitor the side effects of certain types of cancer by ingesting deidentified statements in the regional variety of English language from patients within this region [81,82] is under development. This model could be used to analyze patient responses at a certain point in time for a cross-sectional study or continuously in real time for a long-term longitudinal study to identify the patients in need of care before their scheduled follow-up visit. The ongoing results from this model would be sent to their health care providers for appropriate actions. In case of an emergency, patient-designated community support networks such as religious or other support groups may be intimated to bring the patient to the emergency department so that the patient can receive timely care.

We plan to expand upon our unified informatics platform to integrate programming applications for the development of state-of-the-art applications targeted specifically toward the unmet health care needs of the Appalachian population.

### Conclusions

This paper establishes the value of the Appalachian Informatics Platform in enabling seamless and secure data access, model development through an analytics engine to explore novel and unexpected hypotheses, and simple yet effective communication of all findings via interactive visualization.

The relatively inexpensive nature of such a platform coupled with its demonstrated advantages will hopefully encourage small and midsized rural academic centers, which traditionally have fewer resources than their urban counterparts, to adopt a research informatics platform within their institutions using the template described in this paper as a guide.

## Abbreviations

ACTSI: Appalachian Clinical and Translational Science Institute
CCI: Charlson Comorbidity Index
CDW: clinical data warehouse
CHH: Cabell-Huntington Hospital
ECCC: Edwards Comprehensive Cancer Institute
EHR: electronic health record
ETL: extract, transform, and load
HIPAA: Health Insurance Portability and Accountability Act
MH: Marshall Health
MU JCESOM: Marshall University Joan C Edwards School of Medicine
OLAP: Online Analytical Processing
PHI: protected health information
SQL: structured query language
